# Correlation between transcript profiles and fitness of deletion mutants in anaerobic chemostat cultures of *Saccharomyces cerevisiae*

**DOI:** 10.1099/mic.0.2006/002873-0

**Published:** 2007-03

**Authors:** Siew Leng Tai, Ishtar Snoek, Marijke A. H. Luttik, Marinka J. H. Almering, Michael C. Walsh, Jack T. Pronk, Jean-Marc Daran

**Affiliations:** 1 Department of Biotechnology, Delft University of Technology, Julianalaan 67, 2628 BC Delft, The Netherlands; 2 Institute of Biology Leiden, Leiden University, Leiden, Wassenaarseweg 64, 2333 AL Leiden, The Netherlands; 3 Heineken Supply Chain, Research and Innovation, Burgemeester Smeetsweg 1, 2380 BB Zoeterwoude, The Netherlands

## Abstract

The applicability of transcriptomics for functional genome analysis rests on the assumption that global information on gene function can be inferred from transcriptional regulation patterns. This study investigated whether *Saccharomyces cerevisiae* genes that show a consistently higher transcript level under anaerobic than aerobic conditions do indeed contribute to fitness in the absence of oxygen. Tagged deletion mutants were constructed in 27 *S. cerevisiae* genes that showed a strong and consistent transcriptional upregulation under anaerobic conditions, irrespective of the nature of the growth-limiting nutrient (glucose, ammonia, sulfate or phosphate). Competitive anaerobic chemostat cultivation showed that only five out of the 27 mutants (*eug1*Δ, izh2Δ, *plb2*Δ, ylr413wΔ and yor012wΔ) conferred a significant disadvantage relative to a tagged reference strain. The implications of this study are that: (i) transcriptome analysis has a very limited predictive value for the contribution of individual genes to fitness under specific environmental conditions, and (ii) competitive chemostat cultivation of tagged deletion strains offers an efficient approach to select relevant leads for functional analysis studies.

## INTRODUCTION

While the number of completely sequenced microbial genomes continues to grow explosively, assignment of biochemical and physiological functions to the corresponding genes is progressing at a much slower rate. A case in point is the extensively studied yeast *Saccharomyces cerevisiae*. Ten years after the completion of its genome sequence (Goffeau *et al.*, 1996), 21 % of its genes have neither an experimentally confirmed function nor a function that can be predicted with a high degree of confidence, based on similarity with genes from other organisms (Saccharomyces Genome Database, August 28, 2006; http://www.yeastgenome.org/cache/genomeSnapshot.html) (Hirschman *et al.*, 2006).

Accurate determination of gene function often requires sophisticated and costly experimental techniques. It is therefore worthwhile to select priority targets for functional analysis, via high-throughput methods such as synthetic-lethality screening (Tong *et al.*, 2001, 2004), mapping of physical interaction (Gavin *et al.*, 2002; Krogan *et al.*, 2006) and expression analysis. With respect to the last, DNA microarrays have been extensively used to map genome-wide transcriptional responses to a multitude of environmental parameters (Boer *et al.*, 2003; Causton *et al.*, 2001; Daran-Lapujade *et al.*, 2004; Gasch *et al.*, 2000). This approach yields sets of genes that show common and specific transcriptional responses to individual environmental parameters. The resulting sets of transcriptionally responsive genes often show enrichment for genes with known functions that can be directly correlated with the environmental conditions under study. Additionally, they invariably yield sets of transcripts that encode proteins of unknown function or with a known biochemical function that cannot be readily linked to the conditions studied.

It is generally assumed that, in the case of upregulated transcripts, the biochemical functions of the encoded proteins contribute to the physiological adaptation of the organism to the environmental parameter under study. However, there are few published studies that have systematically investigated the extent to which this concept of ‘transcriptomics-inferred function’ is correct and applicable for guiding functional-analysis research. Two large-scale comparisons suggest that the correlation between transcript profile and fitness of deletion strains may be far from perfect (Birrell *et al.*, 2002; Giaever *et al.*, 2002, 2004; Winzeler *et al.*, 1999).

*S. cerevisiae* is the only yeast that can grow rapidly under aerobic as well as anaerobic conditions (Visser *et al.*, 1990). This unique ability plays a major role in various industrial applications of *S. cerevisiae*, including beer and wine fermentation, and large-scale production of fuel ethanol. Still, the genetic basis for rapid anaerobic yeast growth remains unknown. In a recent chemostat-based study (Tai *et al.*, 2005), we used transcriptome analysis to investigate the response of *S. cerevisiae* to anaerobic conditions. Sixty-five genes (∼1 % of the genome) were found to be significantly upregulated under anaerobic conditions, irrespective of the nature of the growth-limiting nutrient (glucose, ammonium, phosphate or sulfate). In separate experiments with the yeast deletion library (Snoek & Steensma, 2006), 24 genes were shown to be essential for anaerobic (but not for aerobic) growth. Surprisingly, when these two sets of genes, obtained from different experimental approaches, were compared, no overlap was found.

In the present study, we investigate whether genes that are transcriptionally upregulated in anaerobic cultures of *S. cerevisiae* contribute to its fitness under anaerobic conditions. In order to be able to identify subtle effects on fitness, competitive cultivation of a reference strain and a set of null mutants was performed in anaerobic chemostats.

## METHODS

### Strains.

*S. cerevisiae* CEN.PK113-7D (*MAT*a *MAL2-8c SUC2*) (van Dijken *et al.*, 2000) was used as the prototrophic reference strain. All knockout strains (Supplementary Table S1) were constructed in this genetic background. Strains were constructed by using standard yeast media and genetic techniques (Burke *et al.*, 2000). The kanamycin resistance cassette was amplified by PCR with specific primers (Supplementary Table S1) and the pUG6 vector as a template (Guldener *et al.*, 1996). As part of the deletion process, each gene disruption was replaced with a KanMX module and uniquely tagged with two 20-mer sequences (Supplementary Table S1) (http://www-sequence.stanford.edu/group/yeast_deletion_project/deletions3.html). The gene YGR059W was tagged with either a unique downtag sequence or an uptag sequence. The deletion of YOR012W carried along inactivation of the paper and overlapping ORF YOR013W. The double mutant strain yor012WΔ/yor013WΔ will be referred to as yor012WΔ in the rest of the paper. Strains were routinely grown at 30 °C on complex yeast bacto-peptone dextrose (YPD) medium.

### Chemostat cultivation.

Chemostat cultivation was performed at 30 °C in 1 l working volume laboratory fermenters (Applikon) with a stirrer speed of 800 r.p.m., pH 5.0, and a dilution rate (*D*) of 0.10 h^−1^, as described by van den Berg *et al.* (1996). The pH was kept constant, using an ADI 1030 biocontroller (Applikon), via the automatic addition of 2 M KOH. The fermenters were flushed with pure nitrogen gas for anaerobic growth and air for aerobic growth, at a flow rate of 0.5 l min^−1^ using a Brooks 5876 mass-flow controller (Brooks Instruments). The dissolved-oxygen concentration was continuously monitored with an Ingold model 34 100 3002 probe (Mettler Toledo), and was 0 % for anaerobic growth and >70 % for aerobic growth. To sustain anaerobiosis, the vessels of medium were sparged with pure nitrogen gas, and Norprene tubing was used to minimize oxygen diffusion into the fermenters. Anaerobic carbon-limited steady-state chemostat cultures of the reference strain *S. cerevisiae* ygr059wΔ : : uptag (see Results) were grown on a synthetic medium, as described previously (Verduyn *et al.*, 1992). Aerobic carbon-limited chemostat cultures contained the same medium but with 7.5 g glucose l^−1^ and without the anaerobic growth factors Tween-80 and ergosterol. When steady state was achieved, the 30 ml competition mix was aseptically injected into the culture using a syringe. Samples were taken via the effluent line every 24 h for a period of 216 h. The samples were chilled on ice, spun down and frozen at −20 °C for high-molecular-weight DNA extraction.

### Anaerobic batch fermentation.

Anaerobic batch cultivations were performed in 2 l chemostats (Applikon) with a working volume of 1 l. Precultures were grown in mineral medium with 2 % glucose until stationary phase in shake flasks at 200 r.p.m. and 30 °C. Fermenters were inoculated with preculture at OD_660_ 0.1. Cultures were grown in a predefined synthetic medium for anaerobic growth (Tai *et al.*, 2005) with 2 % glucose. pH, temperature and stirrer speed for chemostat anaerobic cultures were maintained as described in the previous paragraph.

### Shake-flask cultivation.

Shake-flask cultivations were performed in 500 ml flasks containing 100 ml medium, which were incubated at 30 °C on an orbital shaker at 200 r.p.m. The composition of the synthetic medium was as follows: 20 g glucose l^−1^, 5 g (NH_4_)_2_SO_4_ l^−1^, 6 g KH_2_PO_4_ l^−1^, 0.5 g MgSO_4_ l^−1^, trace elements and vitamin solutions (Verduyn *et al.*, 1990). The medium was adjusted to pH 5.0 and sterilized by autoclaving. Glucose was autoclaved separately. Vitamins were filter-sterilized and added to the medium. Growth of the various strains was monitored by measuring OD_660_. After growing all strains to mid-exponential phase, an equivalent amount of each mutant strain, corresponding to OD_660_ 0.02, was aseptically pooled to prepare a mixed inoculum (30 ml total volume) for the competition experiments.

### High-molecular-weight DNA extraction.

DNA samples were purified using an adaptation of the method of Burke *et al.* (2000). A volume of 40 ml cell culture broth was spun down and resuspended in 1 ml DNA extraction buffer (2 % Triton X-100, 1 % SDS, 100 mM NaCl, 10 mM Tris, pH 8.0, 1 mM EDTA, pH 8.0). Resuspended cells (400 μl) were added to an equal volume of phenol/chloroform/isoamyl alcohol (25 : 24 : 1), pH 8.0, and 0.3 g sterile glass beads. The Bio 101 Fastprep (Qbiogene) was used to break the cell walls with a speed setting of 4.5 for 15 s. After centrifugation, the supernatant was transferred to 500 μl phenol/chloroform/isoamyl alcohol (25 : 24 : 1), pH 8.0, and vortexed. Supernatant was transferred to 1 ml absolute ethanol (−20 °C) for precipitation of DNA and centrifuged for 15 min (13 000 r.p.m.) at room temperature. The DNA pellet was resuspended in 400 μl Tris/EDTA (TE) buffer (10 mM Tris/HCl, pH 7.4, 1 mM EDTA, pH 8.0) and 15 μl RNase cocktail (Ambion 2286), and kept at 37 °C until fully dissolved. After centrifugation, the chromosomal DNA was reprecipitated with 5 μl 7.5 M ammonium acetate and 1 ml absolute ethanol (−20 °C), and immediately centrifuged at 13 000 r.p.m. for 15 min at room temperature. The air-dried DNA pellet was resuspended in 50 μl TE buffer. Quality of DNA was checked with a 1 % Tris/acetate/EDTA (TAE) agarose gel. DNA quantity was analysed at OD_260_.

### Quantitative real-time PCR (qrtPCR).

qrtPCR was run on a DNA engine Opticon I system (Bio-Rad) with the following settings: 94 °C for 2 min, 94 °C for 10 s, 55 °C for 10 s, 72 °C for 10 s, and plate reading. The denaturation, annealing, elongation and reading steps were repeated for 49 cycles. A melting curve from 55 to 94 °C was performed at the end of the reaction. The reaction mixture of 20 μl consisted of 10 μl SybrGreen TAG readymix (Sigma S1816), 0.2 mM forward primer, 0.2 mM reverse primer and 50 ng DNA. The C(*t*) value was calculated with Opticon Monitor software version 1.08 (Bio-Rad) by setting the threshold for significant detection levels to 10× sd over the cycle range from 1 to 15. Triplicate readings were carried out for each time point.

### Data and statistical analysis.

The C(*t*) values were converted to DNA concentration (*X*_DNA_) via the exponential relationship of *X*_DNA_ and C(*t*): *X*_DNA_=ae^−C(t)^, where a is a constant for each strain the value of which depends on the efficiency of qrtPCR. For each strain, all *X*_DNA_ values measured during the 216 h competition experiment were normalized to the *X*_DNA_ value at *t*=0 to eliminate bias from PCR efficiency. Fitness was calculated by taking the slope of the best-fit linear trend line. The relative reduction of the fitness of mutant strains was calculated from the biomass balance (1):[Disp-formula e001](1)
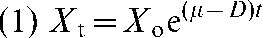
where *t* represents time (h), *X*_t_, biomass concentration at time *t*, *X*_o_, initial biomass concentration, *μ*, growth rate (h^−1^) and *D*, dilution rate (h^−1^). Statistical analysis was done using the modified Z score (Iglewicz & Hoaglin, 1993) to identify mutants that showed a significant reduction in fitness (outliers). The modified Z-score was then subjected to a two-tailed *t* distribution test with two degrees of freedom in accordance with Grubbs' test (Barnett & Lewis, 1994), to calculate the *P* values for each mutant strain. Only mutants with *P*<0.01 were deemed significantly reduced in fitness.

## RESULTS

### Selection of target genes and construction of deletion strains

**Fig. 1. f1:**
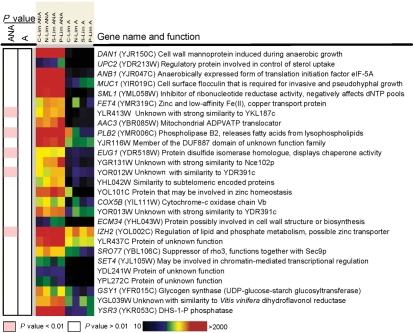
Genes included in the competitive cultivation experiments. Transcript intensities are depicted as a range from low (black) to high (red). Biochemical functions of the encoded proteins are derived from the Yeast Proteome Database (www.proteome.com). *P* values represent the significance of the reduced fitness of the respective mutant strain during aerobic and anaerobic growth. Abbreviations: C, carbon; N, nitrogen; P, phosphorus; S, sulfur; Lim, limited; ANA, anaerobic; A, aerobic; DHS-1-P, dihydrosphingosine-1-phosphate.

A previous transcriptome analysis of *S. cerevisiae* chemostat cultures yielded 65 genes that, irrespective of the growth-limiting macronutrient, showed a higher transcript level in anaerobic chemostat cultures than in aerobic cultures (Tai *et al.*, 2005). For the sake of brevity, we will refer to these genes as ‘anaerobically upregulated’. From these 65 genes, a set of 24 was selected for further analysis (Fig. 1), based on the following criteria. (1) High change in transcript level (more than threefold). This led to the elimination of three genes whose transcript level varied between two- and threefold. (2) Unclear or unknown function. For example, eight of the 65 genes are related to sterol and unsaturated fatty acid metabolism. As these processes require molecular oxygen, their anaerobic upregulation is understood, and we therefore eliminated these genes from the present study. (3) Not part of a family of genes with high sequence similarity. For example, 21 of the 65 anaerobically upregulated genes belong to the seripauperin family (*DAN*, *PAU* and *TIR* genes). Since multiple members of this family were present in the set, redundancy might well have obscured the interpretation of the competitive cultivation experiments carried out with single deletion strains. We therefore decided to eliminate members of large gene families from this study. (4) No previously established clear relation with anaerobic growth.

Five additional genes were selected for inclusion in further experiments. YGR059w was selected as a physiologically neutral marker gene based on transcript data. YGR059w encodes a sporulation-specific septin that functions in cytokinesis, meiosis I and sporulation, and was not expressed in the haploid CEN.PK113-7D strain under 20 different chemostat conditions (see Supplementary Table S2). *URA3*, which is essential for uracil biosynthesis, was included as a negative control: in the absence of uracil, *ura3*Δ strains should not grow. Additionally *DAN1*, *UPC2* and *ANB1* were included as extensively studied, anaerobically upregulated genes. *DAN1* encodes a cell wall mannoprotein induced during anaerobic growth, initially excluded as a member of the seripauperin (PAU) family (Viswanathan *et al.*, 1994). *UPC2* (uptake control 2) encodes a sterol regulatory element binding protein involved in the regulation of sterol biosynthetic gene expression and the uptake and intracellular esterification of sterols (Wilcox *et al.*, 2002). Finally, *ANB1* encodes the translation initiation factor eIF5A that displays specific and strong anaerobic transcriptional upregulation (Wei *et al.*, 1995). In total, 29 genes were further studied by means of competitive cultivation.

### Competitive chemostat experimental design

**Fig. 2. f2:**
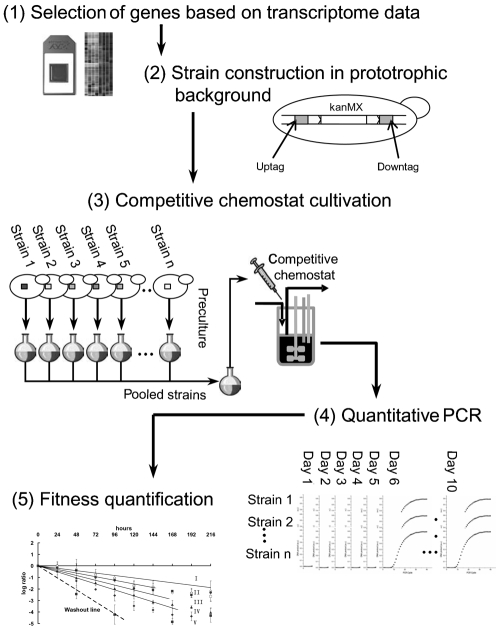
Experimental design. (1) Selection of strains based on their transcript profiles. All the genes tested showed a consistently higher expression in the absence of oxygen than in its presence, with four different nutrient limitations. (2) Knockout mutants of anaerobiosis-induced genes were constructed. Each mutant carried two specific tags. (3) Competitive fermentation: the knockout mutants were grown and pooled in equal proportion prior to injection into a steady-state chemostat culture of the reference strain ygr059wΔ : : uptag. The culture was sampled every 24 h for a period of 9 days. (4) qrtPCR was performed on daily samples, using a tag-corresponding specific primer and a common primer for all strains. (5) Determination of fitness compared to the pooled reference strain ygr059wΔ : : downtag.

An outline of the experimental design is presented in Fig. 2. All 29 genes were deleted from the start to stop codon in *S. cerevisiae* CEN.PK113-7D and replaced with the *kanMX* deletion cassette flanked by two gene-specific 20 nt tag sequences (Winzeler *et al.*, 1999; see Methods). The kanMX cassette has previously been shown not to confer a selective (dis)advantage during prolonged chemostat cultivation of *S. cerevisiae* (Baganz *et al.*, 1997). To further rule out interference by this marker gene we also expressed it in the reference strain.

In contrast to previous large-scale functional-profiling studies (Giaever *et al.*, 2002, 2004; Winzeler *et al.*, 1999) in which auxotrophic mutant collections were screened, all mutants used in this study were generated in the prototrophic CEN.PK113-7D strain (van Dijken *et al.*, 2000). The use of prototrophic strains (with the exception of the *ura3* negative control strain) eliminates the risk that results are influenced by the nutritional requirements of auxotrophic strains (Pronk, 2002).

Subsequently, steady-state chemostat cultures were grown with the neutral control mutant ygr059wΔ containing only the uptag (Fig. 2). A second ygr059wΔ strain carrying a specific downtag sequence was also constructed and added to the mutant pool. This latter strain was used to normalize the population dynamics of the other mutants. The mixture of deletion strains (see Methods) was then injected into the steady-state chemostat culture. We prefer this approach to the inclusion of the mutant pool at the start-up of the chemostat, as reported elsewhere by Baganz *et al.* (1997), when cultivation conditions are dynamic and the selective pressure may differ from that under steady-state conditions.

The culture was then sampled daily over a period of 9 days (216 h). This time frame was chosen to reduce the impact of evolutionary adaptation, which would render a comparison of the fitness of individual tagged mutants impossible (Jansen *et al.*, 2005; Novick & Szilard, 1950) (Fig. 2). After DNA isolation, samples were analysed by qrtPCR, using the molecular tags to monitor the abundance of each mutant. After normalization to the initial sample, the abundance of the deletion strains was normalized to that of the ygr059wΔ : : downtag reference strain included in the mutant pool.

### Competitive anaerobic chemostat cultivation

**Fig. 3. f3:**
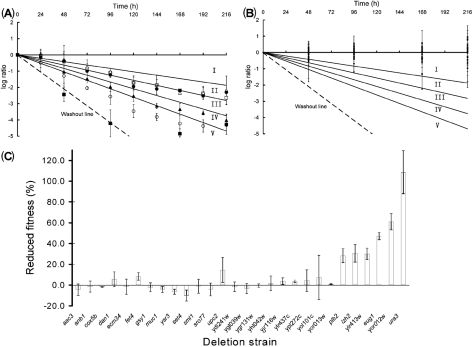
Results of anaerobic competitive chemostat cultures. (A) Strains with fitness reduction: log ratio [ΔC(*t*)_mutant_/ΔC(*t*)_ref_] as a function of time. Graph areas (roman numerals) indicate the following reductions of fitness: (I) <20 %, (II) 20–30 %, (III) 30–40 %, (IV) 40–50 %, (V) >50 %. The dashed line denotes washout (zero specific growth rate). The graph only shows mutants with >20 % reduction of fitness. ▪, *ura3*Δ; □, ylr413wΔ; •, *izh2*Δ; ○, yor012wΔ; ▴, *eug1*Δ; Δ, *plb2*Δ. Error bars indicate mean±sd of two independent chemostat cultures with triplicate measurements for each time point. (B) Strains without fitness reduction: log ratio [ΔC(*t*)_mutant_/ΔC(*t*)_ref_] as a function of time. Error bars indicate mean±sd of two independent chemostat cultures with triplicate measurements for each time point. (C) Bar graph indicating fitness. Reduced fitness of each deletion strain was calculated from the slope of the best-fit linear line. Error bars indicate mean±sd of two independent chemostat cultures.

During the competitive anaerobic chemostat experiments, strains that did not grow (*μ*, 0 h^−1^) were expected to disappear from the culture via washout kinetics at the dilution rate of 0.10 h^−1^. This is depicted by the washout line in Fig. 3(A). Indeed, the auxotrophic *ura3*Δ strain (negative control) closely followed this line (Fig. 3A[Fig f3]). After 96 h, the abundance of the *ura3*Δ strain did not decrease any further (Fig. 3A[Fig f3]). This abundance was taken to reflect the threshold for detection in the experimental set-up. The C(*t*) values measured for the reference strain ygr059wΔ : : downtag did not vary by more than 3.6 % in the duplicate experiments over the period of 216 h.

The anaerobic competitive cultivation experiment was performed in two independent chemostat runs. The fitness of the mutants in the anaerobically upregulated genes observed in these two runs was generally in good agreement (Figs. 1 and 3). The fitness data from each strain were evaluated by means of a statistical test, revealing five outliers (*P*<0.01) from the set of 27 mutants (Fig. 1). Consequently, it was not possible to make reliable statements about decreases in fitness below 20 %. While prolonging the chemostat experiment might have led to increased sensitivity, we decided against this because of the high risk of interference by evolutionary processes (Jansen *et al.*, 2005; Novick & Szilard, 1950).

None of the three anaerobic marker knockout strains *anb1*Δ, *dan1*Δ and *upc2*Δ displayed a significant fitness loss compared to that of the control strain (ygr059wΔ : : downtag). While such a result could be anticipated in the case of *DAN1*, which is part of a large gene family, it was more unexpected in the case of *ANB1* and *UPC2*, which participate in the central processes of transcription and translation. It may be relevant to note that a larger variation in fitness between the two experimental runs was observed for the *upc2*Δ strain than for the *anb1*Δ and *dan1*Δ strains.

Regarding the remaining 24 mutants in anaerobically upregulated genes, only five (*eug1*Δ, *izh2*Δ, *plb2*Δ, ylr413wΔ and yor012wΔ; Fig. 3A[Fig f3]) showed a significant (20–60 %) reduction of fitness in independent replicate experiments (Fig. 3A, C[Fig f3]). Of the five genes whose deletion resulted in a reduction of fitness under anaerobic conditions, *EUG1* is the most extensively documented. *EUG1* encodes a non-essential protein disulfide isomerase (Tachibana & Stevens, 1992). The *S. cerevisiae* genome contains four additional protein disulfide isomerases (*PDI1*, *MPD1*, *MPD2* and *EPS1*), of which only *PDI1* is essential (Norgaard *et al.*, 2001). In addition to their catalytic role in protein folding, protein disulfide isomerases act as chaperones (Kimura *et al.*, 2005). *IZH2/PHO36* has been proposed to be involved in metabolic pathways that regulate lipid and phosphate metabolism (Karpichev *et al.*, 2002). Additionally, *IZH2* is part of the *ZAP1* regulon, and has been proposed to play a role in zinc homeostasis along with *IZH1*, *IZH3* and *IZH4* (Lyons *et al.*, 2004). *PLB2* encodes a lysophospholipase B involved in phospholipid metabolism (Fyrst *et al.*, 1999; Merkel *et al.*, 1999). Two additional lysophospholipase B genes are also found in the *S. cerevisiae* genome: Plb1 (62 % similarity) (Lee *et al.*, 1994) and Plb3 (57 % similarity) (Merkel *et al.*, 1999). The two remaining genes are very poorly characterized. Several experiments indicate that Ylr413wp is localized at the cell surface (Diehn *et al.*, 2000; Huh *et al.*, 2003) but, just like that of YOR012w, its function is totally unknown.

The maximum specific growth rate (*μ*_max_) of the five mutants identified in the competitive experiment was measured in pure culture, using anaerobic fermenters (Table 1). All five exhibited a *μ*_max_ higher than the dilution rate of 0.10 h^−1^ used in the competitive chemostat cultivation. The specific growth rates of the *eug1*Δ, *izh2*Δ and yor012CΔ mutants were significantly lower than that of the reference strain (*t* test analysis, *P*<0.05). However, in none of the mutants did this decrease of *μ*_max_ exceed 18 % (Table 1). Consequently, their reduced competitiveness in the chemostat experiments could not be entirely attributed to a reduced *μ*_max_.

### Aerobic reference experiments

To investigate whether the observed reduction of fitness of the five mutant strains was specific for anaerobic conditions, aerobic competitive chemostat experiments were run. Over a period of 5 days, none of the 27 mutants displayed a significant fitness reduction when compared to the reference ygr059wΔ : : downtag strain (Table 1, Fig. 1). As an additional control, the specific growth rates of the five mutant strains that showed a reduced fitness in the anaerobic cultures were measured in (semi-)aerobic shake-flask cultures, and were found not to differ significantly from those of the isogenic reference strains CEN.PK113-7D and ygr059wΔ : : downtag (Table 1). This implies that the reduction in fitness encountered in five of the mutant strains during anaerobic competitive growth was specific for anaerobiosis.

## DISCUSSION

Previous systematic comparisons of transcript levels and fitness of yeast mutants in batch cultures (Birrell *et al.*, 2002; Giaever *et al.*, 2002, 2004; Winzeler *et al.*, 1999) have used the entire *S. cerevisiae* deletion library. The present study is believed to be the first to use transcriptome data to select target genes in chemostat-based competitive cultivation. We have reported a fitness profiling of knockout strains in genes that showed a consistently higher transcript level under anaerobic conditions than that under aerobic conditions. Our experimental approach differed in several aspects from earlier *S. cerevisiae* (Baganz *et al.*, 1997, 1998; Colson *et al.*, 2004) and *Escherichia coli* (Chao & McBroom, 1985; Dean *et al.*, 1988; Dean, 1989; Trobner & Piechocki, 1985) chemostat-based competition experiments: injection of a mutant pool into a steady-state culture, use of qrtPCR for quantification, and selection of strains based on transcriptome studies. This novel setup was (i) sensitive (qrtPCR has greater sensitivity than quantitative PCR, colony plate counts or Affymetrix tag3 arrays); (ii) cost-effective (goal-orientated gene-deletion selection); and (iii) yielded reproducible results (as determined by the immediate fitness test from steady-state conditions and prototrophic strains). In mixed populations, the possibility cannot be excluded that reduced fitness results from interactions between strains with different genotypes. For example, excretion of a metabolic intermediate by one of the deletion mutants might be toxic to another, or a mutation that is strongly disadvantageous in pure culture may be rescued by cross-feeding by other strains (Pronk, 2002). We sought to minimize the impact of such phenomena by keeping the abundance of each of the mutants in the culture very low.

Our study yielded five priority targets for further functional analysis of the molecular basis for anaerobic growth in *S. cerevisiae*. Further analysis will involve the use of multiple mutations to narrow down gene function. The available literature provides some interesting leads. Lyons *et al.* (2004) have reported that *IZH2* is involved in coordinating both sterol and zinc metabolism under anoxia. The possibility that *izh2* mutants may be impaired in uptake of sterols, which are essential for anaerobic growth of *S. cerevisiae* (Andreassen & Stier, 1953), merits further research. YLR413w encodes a protein of unknown function that has a 49 % sequence similarity to YKL187c, which is transcriptionally upregulated during growth on oleate (Kal *et al.*, 1999). It is conceivable that these genes are implicated in the uptake of essential unsaturated fatty acids, which are essential for anaerobic growth. It is relevant to note that, in the present study, oleate was provided as Tween-80 (polyoxyethylene sorbitan monooleate). Tween-80 was introduced to compensate for the inability of *S. cerevisiae* to synthesize unsaturated fatty acids *de novo* under anaerobic conditions. However, for Tween-80 to act as a source of oleate, the acyl-ester bond that links the oleate chain to the polyoxyethylene sorbitan complex must be cleaved. It is conceivable that this reaction is linked to the loss of fitness recorded for the *plb2*Δ strain. Plb2 may catalyse the hydrolysis of Tween-80 at the single fatty acid ester bond to yield oleate, as it does with lysophosphatidylcholine (Fyrst *et al.*, 1999). The incomplete functional complementation of *PLB1* and *PLB3*, which were also expressed under anaerobic conditions, might then reflect differences in substrate affinity and specificity of all three yeast phospholipases B, as already reported (Merkel *et al.*, 2005).

*EUG1* encodes a protein disulfide isomerase of the endoplasmic reticulum lumen. It has been suggested elsewhere (Ter Linde & Steensma, 2002) that *EUG1* is involved in glycosylation and isomerization of disulfide bonds during the folding of anaerobically synthesized Dan/Tir cell-wall proteins, but this suggestion has not yet been experimentally followed up. The reason for the fitness loss of the yor012WΔ strain, which was actually equivalent to that of the double mutant yor012WΔ/yor013WΔ, remains unknown. As a consequence of the overlap between the ORFs, a more elaborated knock-out strategy should be applied to study each deletion individually and sort out which of the two genes contributes to the reduction of fitness observed.

Of 24 *S. cerevisiae* genes that showed a strong and consistent transcriptional upregulation under anaerobic conditions, but were not previously implicated in anaerobic metabolism, based on other experimental approaches, only five were shown to contribute to fitness under anaerobic conditions, via competitive cultivation of null mutants. At first glance, it might be argued that this low hit rate was due to the low dilution rate in the chemostat cultures (0.1 h^−1^, which is threefold lower than the *μ*_max_ of *S. cerevisiae* CEN.PK113-7D; Kuyper *et al.*, 2004). This interpretation is not correct, however, as mutations that have a negative effect on the maximum specific growth rate will directly affect fitness because they lead to a lower affinity (*μ*_max_ /*K*_s_) for the growth-limiting nutrient (where *K*_s_ is the substrate saturation constant) (Button, 1991; Monod, 1942). Indeed, the *μ*_max_ of five mutants with reduced fitness in anaerobic chemostat conditions differed by <20 % from that of the reference strain. This indicates that a reduced *μ*_max_ was not the sole or predominant cause of the reduced fitness. A similar observation has been made in the bacterium *Ralstonia eutropha*, in which the fitness of a mutant construct cannot be attributed to a reduced *μ*_max_ (Fuchslin *et al.*, 2003). Subsequent kinetic analysis has shown that expression of the *R. eutropha gfp* gene directly affects *K*_s_, resulting in displacement of the *gfp*-expressing strain by the wild-type strain in carbon-limited chemostat cultures (Fuchslin *et al.*, 2003).

Of the five deletion mutants with reduced fitness in anaerobic chemostat cultures, three (*izh2*Δ, *plb2*Δ and *ylr413W*Δ) carry mutations in genes that encode membrane proteins. It is conceivable that these mutations affect membrane structure and thereby the affinity of nutrient-import systems.

Even though we sought to enrich the set of target genes by only including genes that showed a strong and consistent transcriptional upregulation under anaerobic conditions, the low hit rate observed in our study was consistent with two earlier genome-scale comparisons between transcript profiles and fitness, in which *S. cerevisiae* was exposed to DNA-damaging agents (Birrell *et al.*, 2002), and grown under various stress and growth conditions (1 M NaCl, 1.5 M sorbitol, pH 8, and galactose) (Giaever *et al.*, 2002). Our observations show that high transcript levels cannot be interpreted as evidence for unique physiological relevance of the encoded protein under the experimental conditions. This conclusion does not, however, imply that the observed transcriptional upregulation under anaerobic conditions is without biological significance.

Several mechanisms may explain why transcriptional upregulation of a gene is not accompanied by reduced fitness of the corresponding null mutant under the experimental conditions. Functional redundancy is a problem inherent in the analysis of (single) deletion mutants. While we sought to reduce the impact of redundancy by eliminating members of highly related gene families from our study, several of the genes displayed sequence similarity with a single second yeast gene (Fig. 1). For example, the role of the anaerobic ATP/ADP translocase encoded by *AAC3* may well be taken over by its aerobic counterparts Aac1p and/or Aac2p (Drgon *et al.*, 1992). *AAC1* is the only aerobic counterpart, since it is only expressed under aerobic conditions; however, *AAC2*/*PET9*, despite a higher expression in the presence of oxygen, is still expressed under anaerobic conditions (Table 2; Tai *et al.*, 2005). Similar functional complementation could occur for *UPC2* and *ANB1*, since their respective homologues *ECM22* and *HYP2* were expressed irrespective of the oxygen regime (Table 2; Tai *et al.*, 2005).

*FET4* is another anaerobic marker gene. It encodes an Fe(II) low-affinity iron/zinc/copper transport system, and its expression is coregulated by iron and oxygen (Jensen & Culotta, 2002). Under aerobic conditions, iron uptake is mainly achieved through the product of *FET3*, which encodes an Fe(II) high-affinity transport system (Askwith *et al.*, 1996). It is conceivable that deletion of the *FET4* gene was compensated by overexpression of one or more high-affinity transport systems (Table 2). A comparable mechanism of gene-expression autoregulation has already been reported. Upon deletion of *PDC1*, which encodes the major pyruvate decarboxylase, growth on glucose is rescued by overexpression of *PDC5* (Hohmann & Cederberg, 1990). Overall, in *S. cerevisiae*, a quarter of the gene deletions that have no phenotype are compensated by duplicate genes (Gu *et al.*, 2003).

The impact of the upregulation of a gene on fitness may be context dependent. For example, ammonia-limited growth of *S. cerevisiae* leads to coordinated upregulation of transporters and enzymes involved in the assimilation of alternative nitrogen sources, even if these are not available in the growth medium (Boer *et al.*, 2003; Magasanik & Kaiser, 2002; ter Schure *et al.*, 1998). Similar mechanisms may underlie the transcriptional upregulation under anaerobic conditions of some of the genes included in this study. For example, the oxidoreductase encoded by YGL039w may provide an excellent energy-efficient redox sink for anaerobic growth, but only in the presence of its unknown substrate. This would also mean that assessing the contribution of transcriptionally upregulated genes would imply testing strains carrying multiple combinatorial deletions of differentially expressed transcripts.

The implied teleological relationship between transcript profiles and fitness does not necessarily have to exist for all genes that show a consistent transcriptional response to a given stimulus. For example, transcriptional regulation networks may have evolved to couple transcriptional responses to environmental stimuli that tend to coincide in the natural environment. When these stimuli are separated in the laboratory or in industry, not all transcriptional responses have a direct bearing on each individual stimulus.

The present study underlines that, in *S. cerevisiae*, increased transcript levels cannot be interpreted as evidence for a contribution of the encoded protein to the fitness of the cell in the immediate experimental context. A similar conclusion has been drawn based on a comparison of metabolic fluxes and transcript levels of the corresponding genes, which has shown that transcript levels cannot be used as flux indicators (Daran-Lapujade *et al.*, 2004). Rather than diminishing the value of transcriptome analysis, these observations underline the need for integrated systems approaches to understand functions of genes and genomes.

## Supplementary Material

[Supplementary data]

## Figures and Tables

**Table 1. t1:** Anaerobic and aerobic maximal specific growth rate determination The anaerobic-specific growth rates were determined in fully anaerobic controlled batch fermentations. The aerobic-specific growth rates were measured in shake flasks. The right-hand column displays the fitness reduction in aerobic competitive chemostats of the five mutants that showed a significant disadvantage in anaerobic competitive chemostats. The *μ*_max_ data of the mutants were compared to the reference strain (ygr059wΔ) data by means of a *t* test. The significance of the difference is indicated by the *P* value (*P*>0.05). Data are presented as the mean±sd of results from two independent cultures for each strain.

**Deletion mutant**	**Anaerobic batch**		**Aerobic batch (shake flask)**		
	***μ*_max_ (h^−1^)**	***t* test *P* value**	***μ*_max_ (h^−1^)**	***t* test *P* value**	**Fitness reduction (%)**
*plb2*Δ	0.31±0.01	7.05×10^−2^	0.39±0.00	3.99×10^−1^	7.0±3.7
ylr413wΔ	0.25±0.08	3.83×10^−1^	0.38±0.02	6.07×10^−1^	11.6±3.3
*izh2*Δ	0.32±0.00	1.31×10^−2^	0.38±0.01	4.87×10^−1^	15.8±7.4
*eug1*Δ	0.29±0.01	3.42×10^−2^	0.37±0.02	6.38×10^−1^	8.6±0.1
yor012wΔ	0.29±0.00	4.05×10^−3^	0.34±0.00	3.15×10^−3^	14.8±5.6
ygr059wΔ	0.34±0.00	−	0.40±0.02	−	−
CEN.PK 113-7D	0.34±0.01	−	0.39±0.00	−	−

**Table 2. t2:** Transcription intensities of genes with corresponding homologues in anaerobic (ANAe) and aerobic (Ae) chemostat cultures with limitations in carbon (C-Lim), nitrogen (N-Lim), phosphorus (P-Lim) and sulfur (S-Lim) Means±sd derived from three independent chemostat experiments.

**Gene name**	**Transcription intensity (arbitrary Affymetrix hybridisation unit)**							
	**C-Lim ANAe**	**N-Lim ANAe**	**P-Lim ANAe**	**S-Lim ANAe**	**C-Lim Ae**	**N-Lim Ae**	**P-Lim Ae**	**S-Lim Ae**
*AAC3*	355±148	311±71	588±23	387±105	12±0	20±3	21±4	22±7
*AAC1*	60±2	118±15	72±10	103±22	529±76	483±67	440±234	353±26
*AAC2*/*PET9*	803±70	463±34	396±23	364±21	1425±122	1445±47	1478±145	1276±98
*UPC2*	36±25	50±22	90±16	66±15	15±3	12±0	14±3	12±0
*ECM22*	182±58	176±30	164±16	201±33	138±12	152±21	165±20	176±6
*ANB1*	3320±457	2392±254	3193±444	2967±299	25±6	16±3	25±4	18±3
*HYP2*	2534±625	3041±384	3253±505	2695±170	p2985±1161	3547±167	3572±66	3699±496
*FET4*	157±41	334±88	293±19	316±28	12±0	123±30	55±5	17±3
*FET3*	15±4	15±3	13±1	46±23	128±43	29±3	136±19	110±36

## Abbreviations

- qrtPCR, quantitative real-time PCR
