# Supplementary material for: Correlation between transcript profiles and fitness of deletion mutants in anaerobic chemostat cultures of Saccharomyces cerevisiae
Source: Microbiology (Reading). 2007 Mar;153(Pt 3):877–86. doi: 10.1099/mic.0.2006/002873-0 (PMC2895221; doi:10.1099/mic.0.2006/002873-0)
Supplement: [Supplementary data] [file supp_153_3_877__index.html]

 Correlation between transcript profiles and fitness of deletion mutants in anaerobic chemostat cultures of Saccharomyces cerevisiae -- Tai et al. 153 (3): 877 Data Supplement - Supplementary data -- 

## Supplementary data

### Competitive chemostat cultivation of *Saccharomyces cerevisiae* mutants indicates a weak correlation between oxygen-dependent transcriptional regulation and fitness of deletion strains under anaerobic conditions, by S. L. Tai, I. Snoek, M. A. H. Luttik, M. J. H. Almering, M. C. Walsh, J. T. Pronk and J.-M. Daran

*Microbiology* vol. **153**, part 3, pp. 877–886

Supplementary Table S1  [PDF file]  (25 KB)

Supplementary Table S2  [PDF file]  (18 KB)

  
  
**Copyright © 2010 Society for General Microbiology.**
