# Supplementary material for: Correlation between transcript profiles and fitness of deletion mutants in anaerobic chemostat cultures of Saccharomyces cerevisiae
Source: Microbiology (Reading). 2007 Mar;153(Pt 3):877–86. doi: 10.1099/mic.0.2006/002873-0 (PMC2895221; doi:10.1099/mic.0.2006/002873-0)
Supplement: [Supplementary data] [file supp_153_3_877__1.pdf]

Supplementary Table S1. Primers used in this study

KanMX qrtPCR reverse primer (used with uptag primer), TCGATTCGATACTAACGCCGCCATCCAGTG; KanMX qrtPCR forward primer (used with downtag primer), TATTCTGGGCCTCCATGTCGC.

**Gene**	**Forward primer for gene knockout**	**Reverse primer for gene knockout**	**Gene position knockout**	**Knockout size (bp)**	**Uptag for qrtPCR**	**Downtag for qrtPCR**
YGR059W	AAAAGGGAGTCGGTTGTCAACAGACTGTCCTGTCGAATTTCCCAACCCTAGTTCTTAATCTACGGCAGCTGAAGCTTCGTACGC	AGCACTATCTGTGGAATGGCTGTTGGAACTTTTTCCGATTAGGATTGCCCACAAGCGAACAGCATAGGCCACTAGTGGATCTG	Chr 7 from 607576 to 609076	1500	CCCTAGTTCTTAATCTACGG	GGATTGCCCACAAGCGAACA
URA3	TCCTAGTCCTGTTGCTGCCAAGCTATTTAATATCATGCACGAAAAGCAAACAAACTTGTGGGGCACACCCAATTTAGACACAGCTGAAGCTTCGTACGC	ATTTGCAAAGGGAAGGGATGCTAAGGTAGAGGGTGAACGTTACAGAAAAGCAGGCTGGGAAAGGCGCGTCAAGTACAAAGTTAGCATGCCACTAGTGGATCTG	Chr 5 from 116206 to 116936	730	GGGCACACCCAATTTAGACA	GCGCGTCAAGTACAAAGTTA
DAN1	AGTATATTAGCTGTCGCCGCAGCATTAGTGGCAAGTGCAAGTCACGGACTTCTATCACATCAGCTGAAGCTTCGTACGC	ACCAGCAATAGCAGCGGCACCGAAAACACCGTTATTGAACTTGTGAGCCTACTGGATCTCGTGTGCATAGGCCACTAGTGGATCTG	Chr 10 from 708740 to 709603	863	GTCACGGACTTCTATCACAT	GAGCCTACTGGATCTCGTGT
UPC2	CAGAATCACAAGAAAGCGGTGACAAAACCCAGAAGAAGAGAAAAAGTCATCGAGCTAATTGTACTCCGGTTCAGCATGTTCAGCTGAAGCTTCGTACGC	TTTGTTGTTGTCATCGATGGTAATCCGCCACCGAGGAAATCTAGCTAGGAGCTACCAGGACCTTCGCATAGGCCACTAGTGGATCTG	Chr 4 from 889767 to 892468	2701	GTACTCCGGTTCAGCATGTT	TAGGAGCTACCAGGACCTTC
ANB1	ACATACACCTATTTCATTCACACACTAAAACATGTCTGACGGATATTGATCTCACCTGCCTCAGCTGAAGCTTCGTACGC	GCTATAGAAATCTAAAATATCTAATCAGATCTTGGAGCTTCCGCAGATTAGACATCATGCTCGCATAGGCCACTAGTGGATCTG	Chr 10 from 525287 to 524844	443	GATATTGATCTCACCTGCCT	GCAGATTAGACATCATGCTC
MUC1	GGTCCTTTCGCTTCTATTTAACTCGGCTTTGGGTTTTCCAGCCCAGGTAGTACAGTTACGCAGCTGAAGCTTCGTACGC	GGAAGAGCGAGTAGCAACCACATAAAGTTTCCAAGAACCTTGATAGTGCCCGTAAGTCTCCGGCATAGGCCACTAGTGGATCTG	Chr 9 from 393604 to 389582	4022	GCCCAGGTAGTACAGTTACG	ATAGTGCCCGTAAGTCTCCG
SML1	CAAGACTACTTTTACGCTCAAAATCGCTGCCAACAACAACAAGCCCGACTGCTCTTCAGGCTATTCAGCTGAAGCTTCGTACGC	TCCTCGACCTTACCCTGGTTGAACATAGATTTTAGTTCGCCACGGCTTGCTCATAGTATTGCATAGGCCACTAGTGGATCTG	Chr 13 from 159395 to 159684	289	CGACTGCTCTTCAGGCTATT	CACGGCTTGCTCATAGTATT (from YML058C-A)
FET4	AATCCAGGTGCTAGGCCTGACGTTCATCATAGAGCACCTACCGCATCATGGAACATAGACCAGCTGAAGCTTCGTACGC	GGGAAACGCTTTTCTACGTATGATAGAAGTATGCGCCTACCGCCAGATAGTCAAGACGACGCATAGGCCACTAGTGGATCTG	Chr 13 from 914470 to 912942	1528	CCGCATCATGGAACATAGAC	CGCCAGATAGTCAAGACGAC
YLR413W	CCCGATTTGTGCTTTATTGACACTTACAACATTTCTGTTGACCATATACGGACCACAGGCTGCCAGCTGAAGCTTCGTACGC	GGGGCTTTCGTGTTCTTCAATGATTCTTGAGGTAGTAGAGAGTTGAGACCCATTCACATCGCATAGGCCACTAGTGGATCTG	Chr 12 from 951173 to 953150	1977	ATATACGGACCACAGGCTGC	AGTTGAGACCCATTCACATC
AAC3	ATGAGTAGCGACGCTAAGCAACAAGAAACAAACTTTGCCAACAGCTATTAGGACGTGTTGCAGCTGAAGCTTCGTACGC	CGAACAATATCATTTGCAACTGGTCATACATGGAAATAACACATGGTCCGACTGGATCACCTGCATAGGCCACTAGTGGATCTG	Chr 2 from 415977 to 416883	906	ACAGCTATTAGGACGTGTTG	ATGGTCCGACTGGATCACCT
PLB2	CATATTACAGGCTAGCTCGCTAATTTCTGGACTTTCGCTCCACAGCATGAAATTGGTCCACAGCTGAAGCTTCGTACGC	GCTGTTATGGCACCTAACAAACTCATTAGAGTATTAGTGTTCCGCCTAGTGAAATATGTCCAGCATAGGCCACTAGTGGATCTG	Chr 13 from 279628 to 277621	2007	CACAGCATGAAATTGGTCCA	CGCCTAGTGAAATATGTCCA (from YMR007W)

Gene	Forward primer for gene knockout	Reverse primer for gene knockout	Gene position knockout	Knockout size (bp)	Uptag for qrtPCR	Downtag for qrtPCR
YJR116W	GCAATGAACGCCAACTCTACCACGACAGCAATCGGCCTCACCGACTCTACGATACGTCGCTGCAGCTGAAGCTTCGTACGC	TCTAGCTTCGACGTTGGGGCTGGTTTTGCAAGTTTCTTGGGACGTATTGGCATAGTTGCTGCATAGGCCACTAGTGGATCTG	Chr 10 from 640738 to 641576	838	GACTCTACGATACGTCGCTG	GACGTATTGGCATAGTTGCT
YGR131W	GTGCGCATCATAAATGCTGTTTTTCTTATTATATCCATAGGAGAATTTGCACCAAGCGCGACAGCTGAAGCTTCGTACGC	GAAATTGTAGGTATGCCCATTTGTCTTCTTGCCCTCCTTCCGCCGCAAGTATGGTAAATAGCATAGGCCACTAGTGGATCTG	Chr 7 from 754755 to 755245	490	AGAATTTGCACCAAGCGCGA	CGCCGCAAGTATGGTAAATA
YOR012W	AAGAATCCTCTTTGGCGGTAAACTTGACAAGCGATGTAGAGCAGATAATACACTGTCACCCAGCTGAAGCTTCGTACGC	GCATAAAGGTAGTAATAGTCCATTCCCAGAGGCATGACTTCGCCCAAACAAGAGCTTCTGAGCATAGGCCACTAGTGGATCTG	Chr 15 from 356565 to 356898	333	GCAGATAATACACTGTCACC	GCCCAAACAAGAGCTTCTGA
YHL042W	GTAGCAACTGTTATATTCTTCGTCATGAGTATCCGTTCTCCACATACGATCACAGTTAGGTCCAGCTGAAGCTTCGTACGC	CACAGGCAATGTCGGCTTTTCTGATATACGGCAACAATTCCTCGCGGGAACAAGATGCAAGCATAGGCCACTAGTGGATCTG	Chr 8 from 15686 to 16097	229	CATACGATCACAGTTAGGTC	CTCGCGGGAACAAGATGCAA
YOL101C	TTCATTGACTACAATAGAGCAAAGCCCAGTCAAATGCGAGACTATTTCTCACGCCGACAGGGCAGCTGAAGCTTCGTACGC	CGGGCTGATTCAAACTTGAATGCATCAAAATATAAGATTGTGTGTCACCGCACTTCATGGGCATAGGCCACTAGTGGATCTG	Chr 15 from 127874 to 127044	830	TATTTCTCACGCCGACAGGG	TGTGTCACCGCACTTCATGG
COX5B	GCCACATATTCATTGTAAAGAGCATGTATAACAAACACTGATACATCTGCGAAATGCGGCCACAGCTGAAGCTTCGTACGC	CTTCAGATACTCGTCTGATTTCAACTGCCATTCCCTGTTCTCATGCTGTACCATATTGGGGCATAGGCCACTAGTGGATCTG	Chr 9 from 155199 to 155711	512	ACATCTGCGAAATGCGGCCA	TCATGCTGTACCATATTGGG
YOR013W	GGTGAAATTGCTGAGGCAAATGATTTCGATGCAGTCGCAATATGGAGGATAGCTCTCAATTCACCCAGCTGAAGCTTCGTACGC	ATCTTTGTGGTAGAGCATCAAGTAGGCCGTGAATCCCTTCCATGAGTGACAAGGTTCTGAGCATAGGCCACTAGTGGATCTG	Chr 15 from 356762 to 357197	435	AGGATAGCTCTCAATTCACC	CATGAGTGACAAGGTTCTGA
ECM34	CCGCAAAAGTGAAGATGAAAAGAACGAAGCCGCTTTAGCCTACACTCACGACATATAGGTCCAGCTGAAGCTTCGTACGC	GGGTTCTCCTAAATGCTACATGGCAGCTAGAACCATCAACAACATGAGTCCAAAGGTGGCAAGCATAGGCCACTAGTGGATCTG	Chr 8 from 14907 to 15446	539	ACACTCACGACATATAGGTC	CATGAGTCCAAAGGTGGCAA
IZH2	TATTAGAAAGGACTAAGAGTGTGCAAGAGCTGAAGAAGAGGAGACAGGAGCATATTGTACCAGCTGAAGCTTCGTACGC	GACAATCCCGTTCTCCATCTTTATATGGACTAACTCATAACAGCTGATAATTCAAGGCACCGCATAGGCCACTAGTGGATCTG	Chr 15 from 324316 to 323458	858	GAGACAGGAGCATATTGTAC	AGCTGATAATTCAAGGCACC
YLR437C	GGCAAGCAGCCTCGTCCCAAAGAGACAGCTTCAACAACAATGATACTGCCCATACTGGCGCAGCTGAAGCTTCGTACGC	GTCTTCTCTTGGATCCATTAACCATTGTTTCGTGCTCCGCAGGCACCTACGATATGCTCGGCATAGGCCACTAGTGGATCTG	Chr 12 from 1011961 to 1011663	298	TGATACTGCCCATACTGGCG	AGGCACCTACGATATGCTCG
SRO77	TTGAAGAATGTGTCAAATGCTATCAAATCCGCGAGAGTTCAGACCTCAGCTTCAGTAACGCAGCTGAAGCTTCGTACGC	CTCTTCATCATATCCCTTCCCGTTTCTTCCATTGCGTCATACCTTATGTGTAATGAGCGGGCATAGGCCACTAGTGGATCTG	Chr 2 from 13819 to 10902	2917	AGACCTCAGCTTCAGTAACG	ACCTTATGTGTAATGAGCGG
SET4	CACTATCTTCTCGTCATATCAGGCAAGGAAGGACATACACTAGTCCTTCACATATCTGCGCAGCTGAAGCTTCGTACGC	CGTTTATGGCTTACAGTTGTGCATTATTGTATTAAAGTAAGAGATCTAGCCACAGTGTATGCGCATAGGCCACTAGTGGATCTG	Chr 10 from 224989 to 226658	1669	TAGTCCTTCACATATCTGCG	GATCTAGCCACAGTGTATGC
YDL241W	GAAAATGCTCTATTGTTTAAGTGCGGTAGCAAGGGCTATATGGGCACACTAGGAAACTCCACAGCTGAAGCTTCGTACGC	CACTAGCATGAACAGTATGGTAAATTTGGCGAGAGAATGAGGAGACCCACTTAATGGCAACGCATAGGCCACTAGTGGATCTG	Chr 4 from 20647 to 20988	341	GGGCACACTAGGAAACTCCA	GAGACCCACTTAATGGCAAC

**Gene**	**Forward primer for gene knockout**	**Reverse primer for gene knockout**	**Gene position knockout**	**Knockout size (bp)**	**Uptag for qrtPCR**	**Downtag for qrtPCR**
YPL272C	CTCACTTTATCATAATGACTACGTTTAGGCCACTATCAAGTAGACTCAGCCAGATGCCAGCAGCTGAAGCTTCGTACGC	CGTCACCTGCCTATTCAATAAAGGATTCGAAGCATTCTACCAGATGTCAGAATAGCTCACGCATAGGCCACTAGTGGATCTG	Chr 16 from 28139 to 26640	1499	TAGACTCAGCCAGATGCCAG	CAGATGTCAGAATAGCTCAC
GSY1	ATCATCTACTGTTCGAAGTAGCAACAGAAGTAACTAACAGGCCCATCCAGTTGAATGAGAGCAGCTGAAGCTTCGTACGC	CCTCGTAGTATGCAGACGTATCGTTGTCATCATCGTCATTCGTTACATACTCAGCCATAGGCATAGGCCACTAGTGGATCTG	Chr 6 from 176324 to 174304	2020	CCCATCCAGTTGAATGAGAG	CGTTACATACTCAGCCATAG
YGL039W	ACTACTGAAAAAACCGTTGTTTTTGTTTCTGGTGCTACTGGCTAGAAACAGAAGGGTCGTCCAGCTGAAGCTTCGTACGC	CTTCTAGTAATTGCGAGGCAGTATCGACAATGCAATCTCTCACCGATTGTACGATTTGGTGCATAGGCCACTAGTGGATCTG	Chr 7 from 423970 to 424996	1026	CTAGAAACAGAAGGGTCGTC	CACCGATTGTACGATTTGGT
YSR3	TGAATTGGGTGTTACCGAGGACACCATTAAGGTTCAGATGCACTCTATGCAATTAAGGCCCAGCTGAAGCTTCGTACGC	AAGTGAAAAAAACTGGGCATAGCAAAAAGACGGTTGTAGGTATGACAGCACAGGTGGTCCGCATAGGCCACTAGTGGATCTG	Chr 11 from 534861 to 533765	1096	CACTCTATGCAATTAAGGCC	TATGACAGCACAGGTGGTCC
EUG1	TTCAATTTCCAAGACTTGACGTTTCAATTATATGGCAATCAGCAGTGGCCGATTCCCTTTCAGCTGAAGCTTCGTACGC	TATTTTCTTCCAGAAACGATGCGAGTAATCAGATGTTAAACCACGCGAAGAACGGATTTAGCATAGGCCACTAGTGGATCTG	Chr 4 from 1478531 to 1480223	1692	AGCAGTGGCCGATTCCCTTT	CCACGCGAAGAACGGATTTA
