# Supplementary material for: Correlation between transcript profiles and fitness of deletion mutants in anaerobic chemostat cultures of Saccharomyces cerevisiae
Source: Microbiology (Reading). 2007 Mar;153(Pt 3):877–86. doi: 10.1099/mic.0.2006/002873-0 (PMC2895221; doi:10.1099/mic.0.2006/002873-0)
Supplement: [Supplementary data] [file supp_153_3_877__2.pdf]

Supplementary Table S2. Expression data of YGR059W and ACT1 over 20 different chemostat culture conditions at a dilution rate of 0.1 h^{-1}

C source	N source	Aeration	Limitation	YGR059W	*ACT1*	Reference	GEO access no.
Glucose	$(NH_4)_2SO_4$	+ Air	Carbon	12.0±0.0	2628.0±204.9	Tai *et al.* (2005)	GSE1723
Glucose	$(NH_4)_2SO_4$	+ Air	Nitrogen	12.0±0.0	2265.3±106.0	Tai *et al.* (2005)	GSE1723
Glucose	$(NH_4)_2SO_4$	+ Air	Phosphorus	12.0±0.0	2314.4±265.5	Tai *et al.* (2005)	GSE1723
Glucose	$(NH_4)_2SO_4$	+ Air	Sulfur	12.0±0.0	2172.1±249.4	Tai *et al.* (2005)	GSE1723
Glucose	$(NH_4)_2SO_4$	+ 100 % N_2	Carbon	14.8±3.0	3726.5±135.9	Tai *et al.* (2005)	GSE1723
Glucose	$(NH_4)_2SO_4$	+ 100 % N_2	Nitrogen	12.0±0.0	2286.7±362.9	Tai *et al.* (2005)	GSE1723
Glucose	$(NH_4)_2SO_4$	+ 100 % N_2	Phosphorus	16.2±4.0	2516.5±101.4	Tai *et al.* (2005)	GSE1723
Glucose	$(NH_4)_2SO_4$	+ 100 % N_2	Sulfur	12.0±0.0	2345.9±248.9	Tai *et al.* (2005)	GSE1723
Ethanol	$(NH_4)_2SO_4$	+ Air	Carbon	12.0±0.0	3226.6±494.9	Daran-Lapujade *et al.* (2004)	NA
Acetate	$(NH_4)_2SO_4$	+ Air	Carbon	12.0±0.0	3233.9±375.6	Daran-Lapujade *et al.* (2004)	NA
Maltose	$(NH_4)_2SO_4$	+ Air	Carbon	12.0±0.0	4674.3±581.8	Daran-Lapujade *et al.* (2004)	NA
Galactose	$(NH_4)_2SO_4$	+ Air	Carbon	12.0±0.0	2676.4±120.3	Not published	NA
Glucose	$(NH_4)_2SO_4$	+ 100 % CO_2	Carbon	12.3±0.6	2974.6±322.6	Aguilera *et al.* (2005)	NA
Glucose	$(NH_4)_2SO_4$	+ 79 %CO_2 +21 % O_2	Carbon	12.0±0.0	2332.6±219.6	Aguilera *et al.* (2005)	NA
Glucose	$(NH_4)_2SO_4$	+ 79 %CO_2 +21 % O_2	Nitrogen	12.0±0.0	2273.2±225.1	Aguilera *et al.* (2005)	NA
Glucose	Asn	+ Air	Carbon	13.5±2.6	2418.1±122.1	Not published	NA
Glucose	Pro	+ Air	Carbon	12.0±0.0	2294.8±127.6	Not published	NA
Glucose	Phe	+ Air	Carbon	12.0±0.0	2917.2±575.3	Vuralhan *et al.* (2003)	NA
Glucose	Leu	+ Air	Carbon	12.0±0.0	2148.8±204.2	Not published	NA
Glucose	Met	+ Air	Carbon	12.0±0.0	2392.7±143.3	Not published	NA

REFERENCES

Aguilera, J., Petit, T., de Winde, J. H. & Pronk, J. T. (2005). Physiological and genome-wide transcriptional responses of *Saccharomyces cerevisiae* to high carbon dioxide concentrations. *FEMS Yeast Res* **5**, 579–593.

Daran-Lapujade, P., Jansen, M. L., Daran, J. M., Van Gulik, W., de Winde, J. H. & Pronk, J. T. (2004). Role of transcriptional regulation in controlling fluxes in central carbon metabolism of *Saccharomyces cerevisiae*. A chemostat culture study. *J Biol Chem* **279**, 9125–9138.

Tai, S. L., Boer, V. M., Daran-Lapujade, P., Walsh, M. C., de Winde, J. H., Daran, J. M. & Pronk, J. T. (2005). Two-dimensional transcriptome analysis in chemostat cultures: combinatorial effects of oxygen availability and macronutrient limitation in *Saccharomyces cerevisiae*. *J Biol Chem* **280**, 437–447.

Vuralhan, Z., Morais, M. A., Tai, S. L., Piper, M. D. & Pronk, J. T. (2003). Identification and characterization of phenylpyruvate decarboxylase genes in Saccharomyces cerevisiae. *Appl Environ Microbiol* **69**, 4534-4541.
